# Regulation of transcriptome networks that mediate ginsenoside biosynthesis by essential ecological factors

**DOI:** 10.1371/journal.pone.0290163

**Published:** 2023-08-17

**Authors:** Zhongce Wang, Zhiguo Chen, You Tang, Meiping Zhang, Meng Huang

**Affiliations:** 1 College of Electrical and Information Engineering, Jilin Agricultural Science and Technology University, Jilin, Jilin, China; 2 College of Information and Control Engineering, Jilin Institute of Chemical Technology, Jilin, Jilin, China; 3 College of Life Sciences, Jilin Agricultural University, Changchun, Jilin, China; Macau University of Science and Technology, MACAO

## Abstract

Ginseng, a valuable Chinese medicinal herb, is renowned worldwide for its effectiveness in alleviating certain conditions and promoting overall health. In this study, we performed weighted gene co-expression network analysis (WGCNA) on the accumulation of essential saponins under the influence of 13 essential environmental factors (including air temperature, air bottom temperature, surface mean temperature, soil temperature, surface shortwave radiation, soil moisture, soil water content, rainfall, total precipitation, elevation, soil type, soil pH, and soil water potential). We identified a total of 40 transcript modules associated with typical environmental factors and the accumulation of essential saponins. Among these, 18 modules were closely related to the influence of typical environmental factors, whereas 22 modules were closely related to the accumulation of essential saponins. These results were verified by examining the transcriptome, saponin contents, environmental factor information and the published data and revealed the regulatory basis of saponin accumulation at the transcriptome level under the influence of essential environmental factors. We proposed a working model of saponin accumulation mediated by the transcriptional regulatory network that is affected by typical environmental factors. An isomorphic white-box neural network was constructed based on this model and the predicted results of the white-box neural network correlated with saponin accumulation. The effectiveness of our correlation-directed graph in predicting saponin contents was verified by bioinformatics analysis based on results obtained in this study and transcripts known to affect the biosynthesis of saponin Rb1. The directed graph represents a useful tool for manipulating saponin biosynthesis while considering the influence of essential environmental factors in ginseng and other medicinal plants.

## Introduction

In plants, the investigation of how environmental factors affect the production of secondary metabolites is an ongoing pursuit due to their close relationships with plant growth and development [1]. Plant secondary metabolites are usually defense compounds responding to various biotic and abiotic stresses [2]. Secondary metabolites produced by medicinal plants are important beneficial compounds for many reasons, and their effects on human health have garnered significant attention [3]. Among these, ginsenosides from *Panax* ginseng, a traditional Chinese medicine, are of particular interest due to their health-promoting and disease-alleviating properties [4–6]. Ginsenoside Rb1, a highly valued natural compound, has garnered extensive attention and investigation for its diverse range of biological activities and medicinal properties. This compound plays a pivotal role in various physiological processes, such as safeguarding the cardiovascular system [7–10], regulating the central nervous system [11–16], modulating immune responses [17–20], enhancing skeletal health [21], promoting metabolism [22], and exhibiting anti-diabetic effects [17]. Notably, ginsenoside Rg1 has been shown to prevent depression-like behaviors and neuronal structural plasticity induced by chronic stress [23].

Understanding the relationship between the biosynthesis of ginsenosides and environmental factors has been a hot research area, and recent developments in transcriptome analysis have brought about new opportunities for such studies. Many studies have explored how environmental factors affect ginsenoside accumulation. However, it is still unclear how the expression of certain genes (including *CYP716A52v2*, *CYP716A53v2*, *DS*, *β-AS*, and *PgSE2*) can affect the accumulation of certain secondary metabolites under the influences of environmental factors. Park et al. reported the effects of overexpression and transcriptome interference of the ginsenoside biosynthesis gene CYP716A53v2 on the composition of ginsenosides [24]. The key enzyme Squalene Synthase (SS), which mediates the biosynthesis of ginsenosides, was cloned by Liang et al. [25]. Han et al. investigated the regulation of ginsenoside and phytosterol biosynthesis by RNA interferences of squalene epoxidase genes (PgSQE1 and PgSQE2) [26], and Kim et al. analyzed ginsenoside profiles and the expression of related genes in *Panax* ginseng Meyer [27].

The relationship between typical saponin content and environmental factors was also examined at the macro level. For example, Chen et al. analyzed the correlation between ginsenoside content and ecological factors in *Panax* ginseng [28]; Hou et al. studied how ecological factors affect the contents of secondary metabolites in medicinal plants including Illumination, photo quality, temperature, moisture and soil factor (soil texture, soil mass elements, soil trace elements and soil microorganism) [29], and Jia et al. specifically investigated the correlation between ginsenoside content and ecological factors in ginseng [30].

Previous research has mainly explored the association between gene regulatory networks and saponin accumulation at a local mesoscopic level. This approach has revealed intricate relationships among environmental factors, transcriptome expression profiles, and saponin accumulation and spatial–temporal characteristics at the macro and micro levels. Wang et al. investigated the correlation between ginsenoside content in 15 tissues and the expression of six ginsenoside biosynthesis-related genes [31]. Other studies have mainly examined the effects of relevant enzymes on saponin accumulation and the relationship between saponin biosynthesis regulatory networks and saponin accumulation [24–26, 32–41]. Thus, investigators have examined saponin biosynthesis at macro, micro, and local mesoscopic levels. Nonetheless, few integrative studies have explored the relationships among transcriptional regulatory networks, environmental factors, and saponin accumulation at the mesoscopic level.

In this study, we explored the relationships between the ginseng transcriptome and the accumulation of essential saponins under the influence of essential environmental factors. Using weighted gene co-expression network analysis (WGCNA), we constructed a transcriptome expression correlation matrix based on known data. We identified a set of highly correlated transcripts that were significantly associated with the accumulation of essential saponins. Our findings offer new insights into the regulatory mechanisms of ginsenoside biosynthesis and provide a basis for further studies on the interactions between environmental factors and metabolite accumulation.

## Materials and methods

### Data sources and data pre-processing

**Fig 1:**
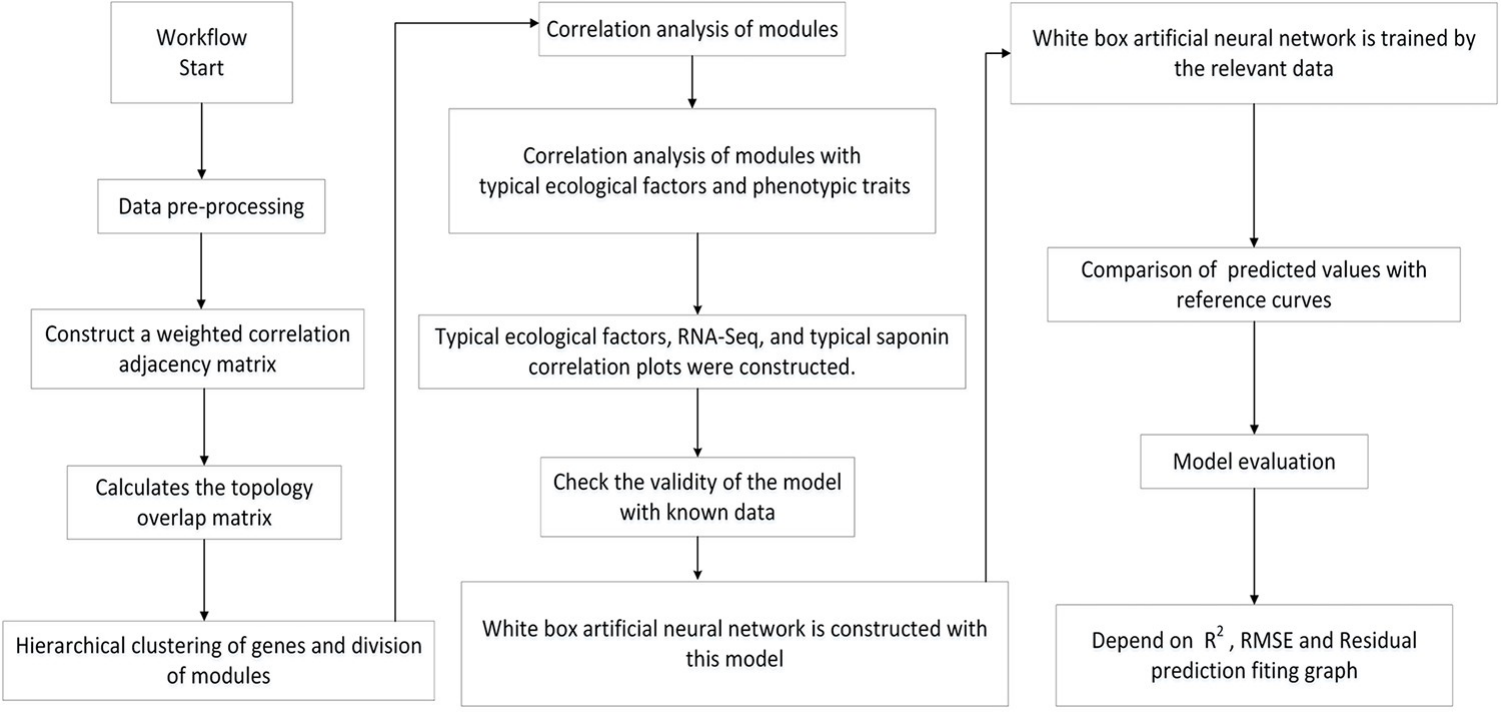
Workflow of the study.

Transcriptome data and saponin contents in samples collected from seven different ginseng-growing regions in Jilin Province, China, at different growth stages, were provided by the Jilin Province Ginseng Gene Resource Development and Utilization Engineering Research Center. Climate data were obtained from the Chinese National Meteorological Data Center (CRA40LAND, China Global Land Surface Reanalysis 40-year Product). The transcriptome sequencing data corresponded to 42 ginseng samples, each composed of 14 tissues. Samples with low expression levels were filtered out. Refer to Fig 1 for workflow of the study (refer to S1 Fig for a more detailed workflow).

The growth stages of ginseng can be classified as the vigorous growth stage, normal growth stage, and aging stage (reference). The first to fourth years of ginseng growth, which are defined as the vigorous growth stage, are characterized by longitudinal and root growth. Longitudinal growth rate of the second year is 300%–400% higher than that of the first year, growth rate of the third year is 200% higher than that of the second year, and growth rate of the fourth year is 100% higher than that of the third year. During the 5–8 year period, the root system exhibits a normal growth rate―the rate at 5–6 years is about 90%–50% of that at 4 years, and the rate at 7–8 years is about 30%–20% of that at 5–6 years. This stage is characterized by lateral root growth. The aging stage occurs after the ninth year, and the growth rate of the ninth year is only about 10% higher than that of the eighth year [42]. At the fourth year, ginseng undergoes a transition from vigorous growth to normal growth, which involves both vertical and lateral root growth, and roots of the plant have more functional activity. At this stage, the response of ginseng to changes in external ecological factors is more comprehensive, and saponin accumulation increases rapidly, which better reflects the response of saponin accumulation for ginseng to environmental factors. Therefore, most analyses in this study were carried out on fourth-year ginseng, and ginseng samples from other age groups were also included for model training and further data analysis.

We sequenced the mRNAs extracted from 14 different tissues of four-year-old ginseng plants using the Illumina HiSeq^TM^2000 with a 100-nucleotide PE (paired-end) module. These 14 tissues were fiber root, leg root, main root epidermis, main root cortex, rhizome, arm root, stem, leaf peduncle, leaflet pedicel, leaf blade, fruit peduncle, fruit pedicel, fruit flesh, and seed. Clean reads were extracted from raw reads using the SOAPnuke1.5.0 software (https://github.com/ BGI-flexlab/SOAPnuke) by BGI (Hong Kong, China) [43] by removing the adapters and low-quality reads (Q ≤ 10, P < 0.05). The clean reads were used to assemble unigenes using the Trinity software [44, 45], and quantify the expressions of individual transcripts using the RSEM (RNA-seq by Expectation Maximization) software [56]. The transcriptome assembled using all clean reads from the 14 tissues was used as the reference (S1 Table).

### WGCNA analysis

The WGCNA package (version 1.70–3) in R (https://cran.rstudio.com/ bin/windows/base version 4.0.5) was downloaded using the BiocManager package (version 1.30.10) and used to construct a co-expression network for transcriptome analysis [46, 47]. First, the goodSamples-Genes function in WGCNA was used to filter the expression matrix and remove unsatisfying transcripts and samples that contained missing data or exhibited zero variance. Second, sample clustering analysis was performed using the flashClust tool in R to detect outliers. Third, pairwise comparisons of transcripts were performed by calculating the Pearson correlation coefficient (PCC) of the expression matrix. Finally, the appropriate soft threshold (β) was selected using the pickSoftThreshold function to ensure the generation of a scale-free network. The adjacency matrix was then constructed using the power function:

$$a_{ij}={\left|S_{ij}\right|}^{\beta }$$

where *S*_*ij*_ represents the Pearson correlation coefficient between transcripts *i* and *j*, and *a*_*ij*_ represents the adjacency relationship between the two transcripts. The topological overlap matrix (TOM) was constructed using the adjacency function:

$$TOM_{\mathrm{ij}}=\frac{\sum_{u}a_{iu}a_{uj}+a_{ij}}{\min(k_{i}{,k}_{j})+1-a_{ij}}$$

where $\sum_{u}a_{iu}a_{uj}$ is the product’s sum of the adjacency coefficients of the nodes connected by transcripts *i* and *j*, and *k* is the sum of the adjacency coefficients between the given transcript and all other transcripts in the weighted network. TOM is used to calculate the dissimilarity measure (1-TOM), and transcripts are assigned to modules using the dynamic cut tree method [48] based on the similarity of expression profiles [49]. The minimum number of transcripts per co-express module was set to 30.

We identified transcript co-expression modules (TCEMs) that exhibited expression patterns similar to those associated with the 13 essential ecological factors and the contents of essential saponins including Rb1, Rb2, Rb3, Rc, Rd, Re, Rf, Rg1, Rg2, and total saponins (TS) determined by liquid chromatography [50]. First, principal component analysis was used to describe featured gene (eigengene) by the first principal component in each module, which corresponded to unified featured gene expression profile within each module. The module membership (MM) corresponds to the correlation between the featured gene and each individual transcript within the same module. Second, gene significance (GS) was assigned to the linear relationship between transcripts expression profiles of specific module and the phenotypes (saponins profiles and environmental factors), which was expressed as logarithm of the P-value of the individual transcript significance. If the GS was stably correlated with the module membership (MM), then it was defined as the correlation between the module featured gene (eigengene) and the phenotype.Finally, we constructed a directed graph reflecting the relationship between ecological factors and saponin accumulation.

To ensure the validity of the results, the related directed graph, which was required to use published methods and data, was tested to determine whether it was compatible with existing knowledge in the field. The effectiveness of the network structure model was assessed using saponin Rb1. The correlations between known transcripts related to saponin Rb1 contents and TCEMs identified by the directed graph were validated. Transcripts possibly related to saponin Rb1 biosynthesis were determined by Gene Ontology analysis [51], and the distribution of co-expression modules in the directed graph was studied using bioinformatics methods. The compatibility of the related directed graph with the existing knowledge in the field was further tested by examining the relative position of corresponding modules in the directed graph and their association with the saponin Rb1 node.

Validity of the combination of every non-associated module for effective saponin synthesis was verified using the chi-square method. First, we set the null hypothesis H0, where the number of different combination categories was unrelated to the effectiveness of Rb1 synthesis. Second, we determined the degrees of freedom (DF) using the following equation:

DF=(a−1)(b−1) (1)

where *a* and *b* are the numbers of categories corresponding to two different test conditions (the number of TCEMs combination categories and combinations of the target effect), with *a* representing the number of combination categories of related or unrelated TCEMs, and *b* representing the number of frequency point categories related or not related to the target effect. The selected significance level α was 0.05. Third, the experimental conditions NAMC and nNAMC are the number of combinations of associated and unassociated modules calculated based on the transcript number of relevant modules, respectively. The initial observation data for the contingency table were constructed, and the theoretical (expected) frequency of the chi-square was calculated. Finally, the chi-square analysis was performed, and P values were calculated. Based on the results of chi-square analysis, the dependency of the number of different combinatorial categories on the effectiveness of identifying ginsenoside Rb1 biosynthesis-related transcripts was determined.

In this study, a white-box neural network model was constructed based on a directed graph. Unlike traditional neural networks, this white-box model had an explicitly interpretable biological structure. The model was trained using typical ecological factors and published data of saponin accumulation in samples of the same developmental stages [52]. The white-box neural network model simulated the accumulation of essential saponins under specific ecological factors, and its consistency was validated with known cases.

## Results

### Data pre-processing

**Fig 2:**
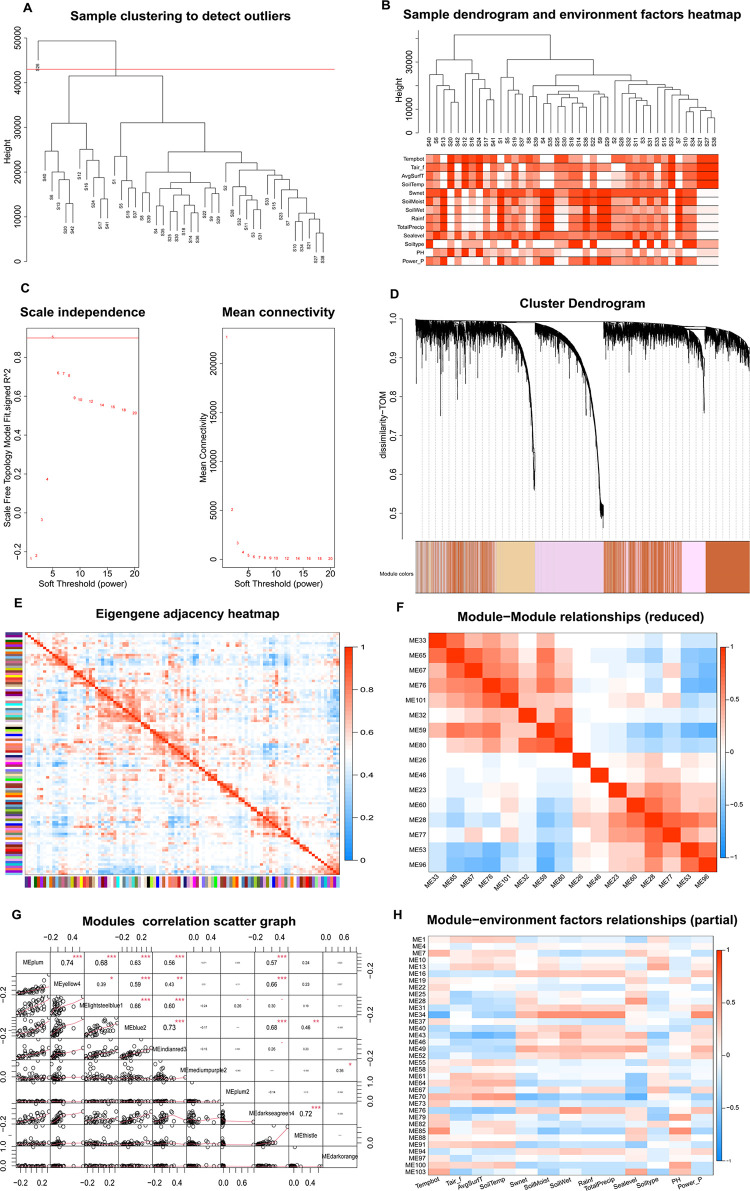
Weighted gene co-expression network analysis (WGCNA). (A) Clustering dendrogram of samples based on their Euclidean distance for identifying outliers. (B) Sample dendrogram and environment factor heatmap. Using the association analysis of effective samples and corresponding environmental factors, the correlations between the similarity of clustered sample features and the change rule of environmental factors provide the basic law for the subsequent in-depth analysis. (C) Soft threshold values were enumerated to determine whether the R^2^ value characteristic of scale-free distribution was met, so as to obtain the best candidate soft threshold value (β), and the corresponding degree of average connection was combined to confirm this value. (D) Dendrogram of transcripts that shows the co-expression modules defined by WGCNA (designated by different colors). (E) Visualization of the eigengene network that represents the relationships among the TCEMs weight. (F) Correlations among transcripts aggregating within modules. (G) Combination of the scatter plot, correlation coefficients, and significance levels.*, P < 0.05; **, P < 0.01; ***, P < 0.001. (H) Correlation between modules and essential environmental factors.

A total of 248,993 mRNA-seq data points in each ginseng sample were used for subsequent analyses. After filtering out samples that contained missing data or zero variance, 73,640 data points were removed. Using the Euclidean distance method, the samples were clustered into two groups, and hierarchical clustering was performed, with all samples clustering under a critical height threshold (43000), except for an outlier (S26), which was identified and removed (Fig 2A).

### Identification of co-expression modules by the WGCNA

Based on an expression matrix composed of 175,353 mRNA-seq data points, ginsenoside profiles, and environmental factors, a network was constructed using WGCNA, and clustering analysis was performed to check the quality of the data obtained from the 42 ginseng samples. These correlations were confirmed by analyses of hierarchical clustering, heat maps, and adjacency relationships (Fig 2A–2E). In terms of ginsenoside accumulation, the featured gene of module ME7 was positively correlated with Re (Rcor = 0.62, P = 2 × 10^−5^), whereas that of module ME12 was positively correlated with Rf (Rcor = 0.68, P = 9 × 10^−7^) (S4 Fig). Thirteen climate variables were applied in WGCNA (Fig 2B). To create a scale-free network, the soft threshold β was set to 5, with a decision coefficient of 0.9 and an average connectivity close to 0 (Fig 2C). TCEMs exhibiting similar expression patterns were grouped into the same module, and modules with a height difference less than 0.25 were merged. This process yielded 103 co-expression modules, with the gray module containing transcripts that could not be assigned to any other module. Some of the modules exhibited stable correlations between their featured genes and other modules (Fig 2F, 2G) or environmental factors. For example, temperature had a significant impact on ginseng growth, with the featured gene of module ME29 negatively correlated with the average surface temperature (Rcor = -0.62, P = 2 × 10^−5^), whereas the featured gene of module ME69 was positively correlated with this environmental factor (Rcor = 0.66, P = 2 × 10^−6^). In addition, ginseng growth was highly dependent on altitude, with the characteristic genes of module ME29 positively correlated with elevation (Rcor = 0.63, P = 9 × 10^−6^). Soil pH was also closely related to ginseng growth, with the featured gene of module ME85 positively correlated with soil pH (Rcor = 0.53, P = 3 × 10^−4^) (Fig 2H, S2 and S3 Figs). Our analysis indicates that some modules may be closely associated with essential environmental factors, whereas others may promote ginsenoside accumulation. As associations may be direct or indirect, further analysis is required to understand the relationship between these modules and ginsenoside content.

### Relationships among essential ecological factors, saponin types, and co-expression modules

**Fig 3:**
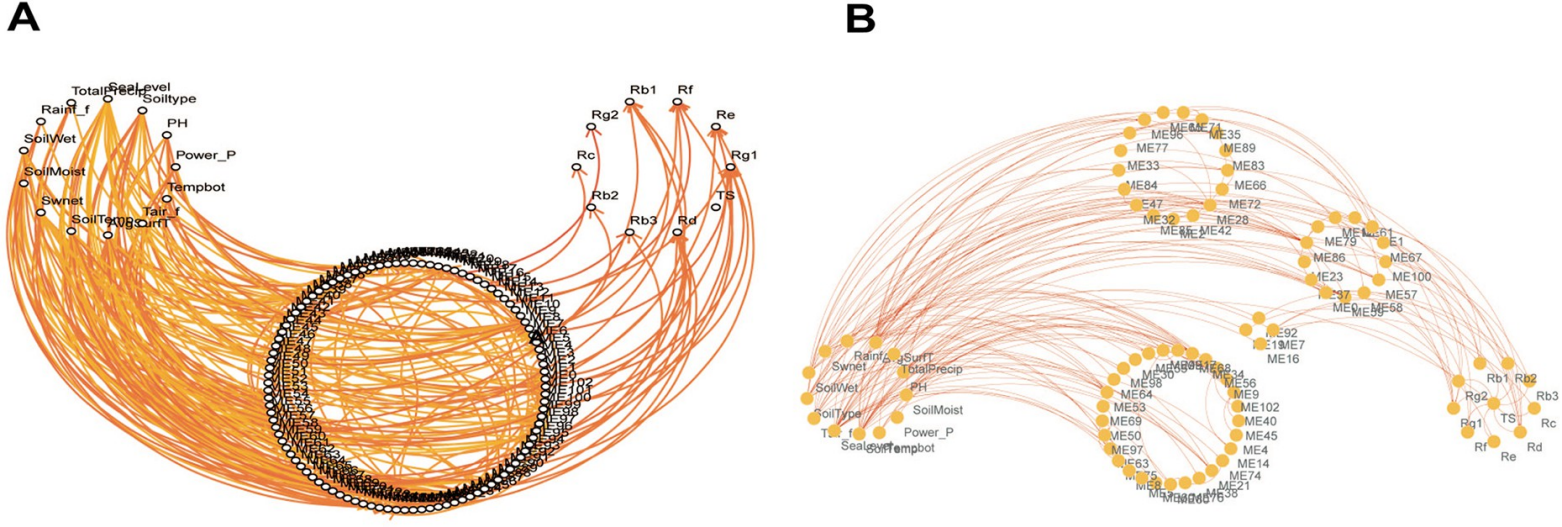
Association diagram of ecological factors, saponin profiles, and TCEMs. (A) Correlations of ecological factors and essential saponins with TCEMs. (B) Correlations among ecological factors, essential saponins, and grouped TCEMs.

In this study, the relationships among essential ecological factors, saponin types, and co-expression modules were examined. For each ecological factor, the correlation coefficient served as a guide to construct a directed graph that connected any two nodes corresponding to the current node and successor node (typical ecological factor, TCEM or saponin type) with the correlation coefficient as the edge weight (Fig 2F–2H). In addition, the layer-by-layer exploration method was used to push forward the correlation network until the corresponding typical saponin node was reached, yielding a data-related directed graph of typical environmental factors, transcriptional regulatory networks, and saponin accumulation. Based on stable biological correlation properties among modules, we postulated that many changes in transcript levels were the result of combined action of multiple environmental factors. By factor analysis, the cumulative contribution rate of the variance between the precursor node and the current module was greater than 80%, which served as the threshold for filtering edges. Candidate precursor nodes were sorted in descending order of contribution of variance. On the other hand, an edge with an absolute correlation coefficient greater than 0.4 between the two nodes corresponding to the current node and successor node was used as another candidate condition. The ecological factors, transcriptional regulatory network nodes, and saponin trait fusion networks were macroscopic in expression (Fig 3A). Further indirect and direct relationships between environmental factors and essential saponins have been obtained through the visualization tool Cytoscape (Fig 3B).

### Positive test

**Fig 4:**
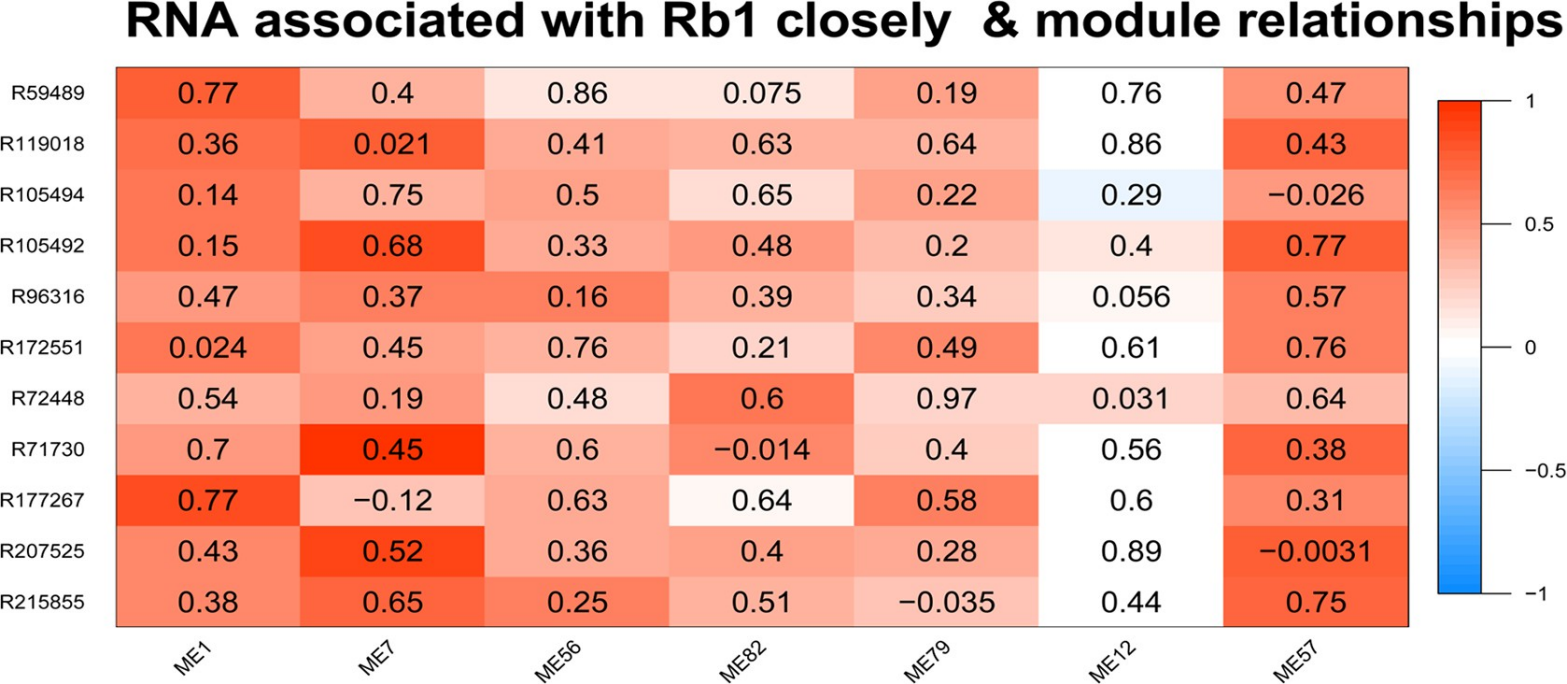
Transcripts closely associated with Rb1 and different modules.

**Fig 5:**
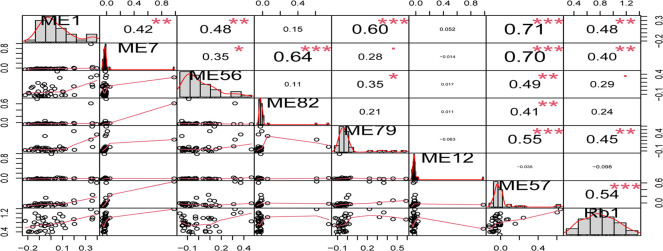
Correlation coefficient, significance level, and corresponding sample scatter plot of transcripts closely associated with Rb1 and different modules. *, P < 0.05; **, P < 0.01; ***, P < 0.001.

Genes that were recognized as closely related to saponin Rb1 biosynthesis were used to test the effectiveness of the model (Table 1). In the positive test, we validated the correlation between the directed graph of saponin biosynthesis regulatory networks affected by environmental factors and the transcripts closely corresponding to saponin Rb1 biosynthesis. The results clearly showed a direct correlation between the transcript clusters corresponding to saponin Rb1 and TCEMs including these associated transcripts with saponin Rb1 biosynthesis (Figs 4 and 5). In addition, indirectly associated modules that are consistent with general domain knowledge should also be considered.

**Table 1:** Corresponding transcript levels of genes related to Rb1 biosynthesis in different samples.

RNA no.	Gene name	RNA transcriptome expression levels (TPM^a^)				
		Sample ID (partial)				
		S1	S2	S3	S4	S5
R59489	*CYP716A53v2_1 (PgCYP137)*	75.46	34.91	26.16	95.97	46.3
R119018	*CYP716A52v2_3 (PgCYP311)*	62.21	15.2	32.65	78.32	24.22
R105494	*DS* *_* *1*	4.68	8.23	6.23	33.16	23.61
R105492	*DS* *_* *3*	53.58	5.34	5.74	22.35	15.69
R96316	*CYP716A47 1 (PgCYP339)*	17.42	10.16	5.09	25.07	15.07
R172551	*β-AS* *_* *1*	4.37	0	4.57	0.84	0
R72448	*SS* *_* *1*	57.97	17.85	25.64	47.21	86.91
R71730	*PgSE2* *_* *1*	0.99	3.49	0	3.22	1.68
R177267	*PgSE2* *_* *4*	55.45	15.3	17.07	71.99	21.71
R207525	*FPS* *_* *22*	62.77	51.93	41.84	68.5	40.72
R215855	*PgUGT71A27* *_* *2*	93.83	86.79	63.09	144.09	46.63

^a^Transcripts per kilobase per million mapped reads

**Fig 6:**
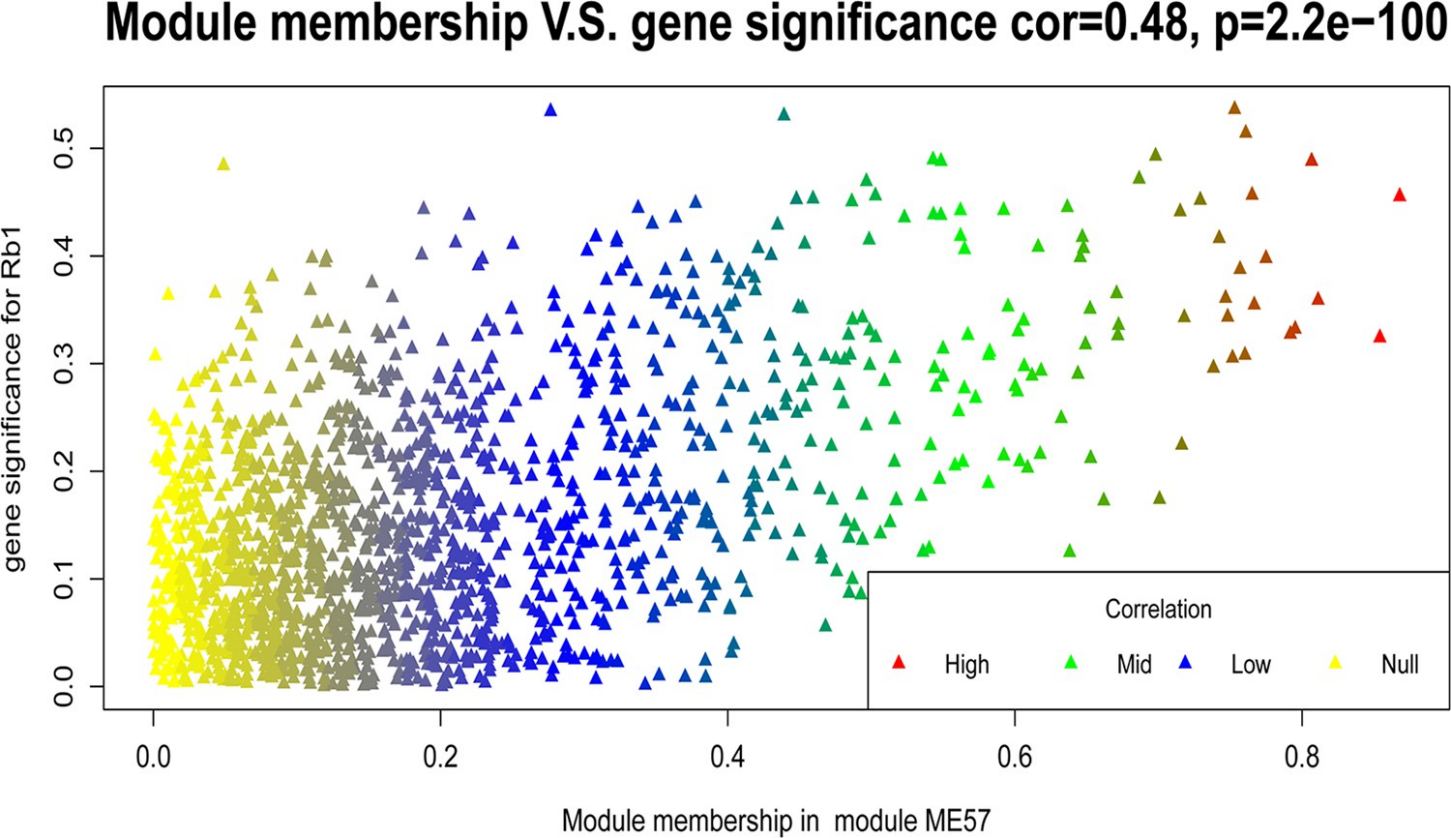
Scatter plot of gene significance for Rb1 versus module membership in module ME57.

The association between GS and MM of module ME57 was examined (Fig 6). The scatter plot shows the consistency of the statistical characteristics of ME57 module transcripts with transcripts closely related to saponin Rb1. This graph also revealed other transcripts that were closely related to the biosynthesis of saponin Rb1, such as those in the upper right corner of the module, providing references for future research. In addition, the statistical characteristics of ME57 module transcripts showed a stable correlation between the GS value of the saponin Rb1 profiles and the MM value of the ME57 module transcripts. As shown in Fig 6, the transcripts closely related to the module (upper right corner) were also closely related to saponin Rb1 profiles, whereas transcripts not closely related to the module (lower left corner) were also not related to the trait.

**Fig 7:**
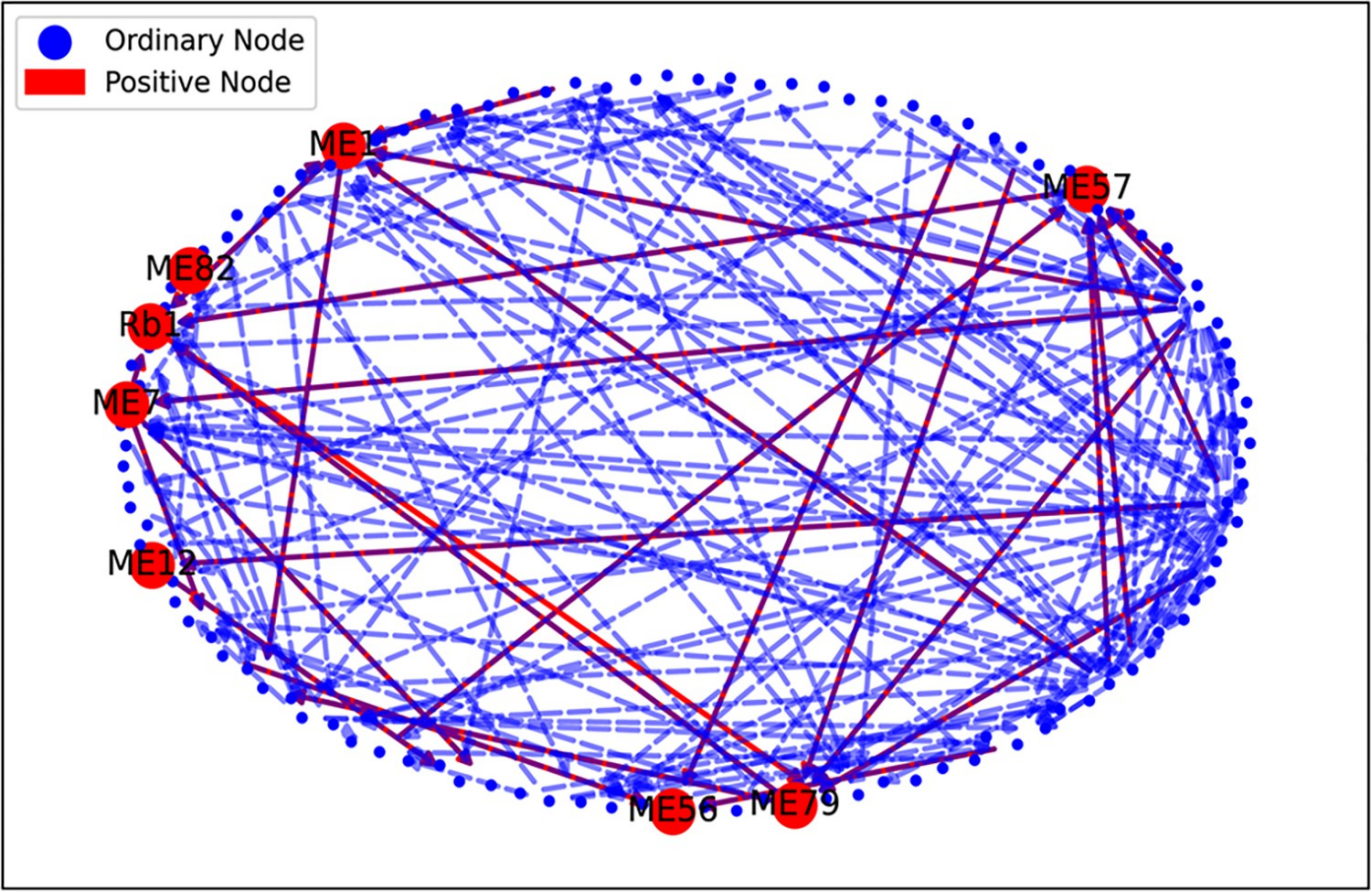
Overlap between transcripts known to influence saponin Rb1 biosynthesis and those from the constructed network. Ordinary node is one that contained in directed graph, and positive node is the transcriptome co-expression module including transcripts that are closely related to saponin Rb1.

The statistical characteristics of module ME57 transcripts reflect its biological significance (Fig 6). The homogeneity between the transcriptional regulatory network, ecological factors, saponin profiles, and Rb1-related transcripts is shown in Fig 7. The coverage of correlations between some nodes and edges of the constructed directed graph and several nodes and edges related to saponin Rb1 biosynthesis reflects the ability of the model to interpret Rb1-related data.

### Negative test

Chi-square analysis was used for negative validation. The null hypothesis was that the number of different combination categories was independent of the combination of TCEMs related closely to Rb1 biosynthesis. Next, the DF value was calculated (formula 1), and a contingency table was constructed (Table 2).

**Table 2:** Validity of saponin biosynthesis determined by the chi-square test.

Combined categories	Frequency	Effective frequency	Invalid frequency	Total
NAMC^a^	86.28	86.28	0	86.28
nNAMC^b^	6684.53	0	6684.53	6684.53
Total	6684.53	86.28	6684.53	6684.53

^a^Number of associated module combinations (NAMC)

^b^No direct correlation with the number of associated module combinations (nNAMC)

Chi-square analysis showed X^2^ = 6691.56, DF = 1, and P < 0.01, indicating that the null hypothesis (H0) was not valid, and the different combinatorial types were related to the efficiency of saponin Rb1 biosynthesis. The model’s scientific rigor was verified through both positive and negative tests.

**Fig 8:**
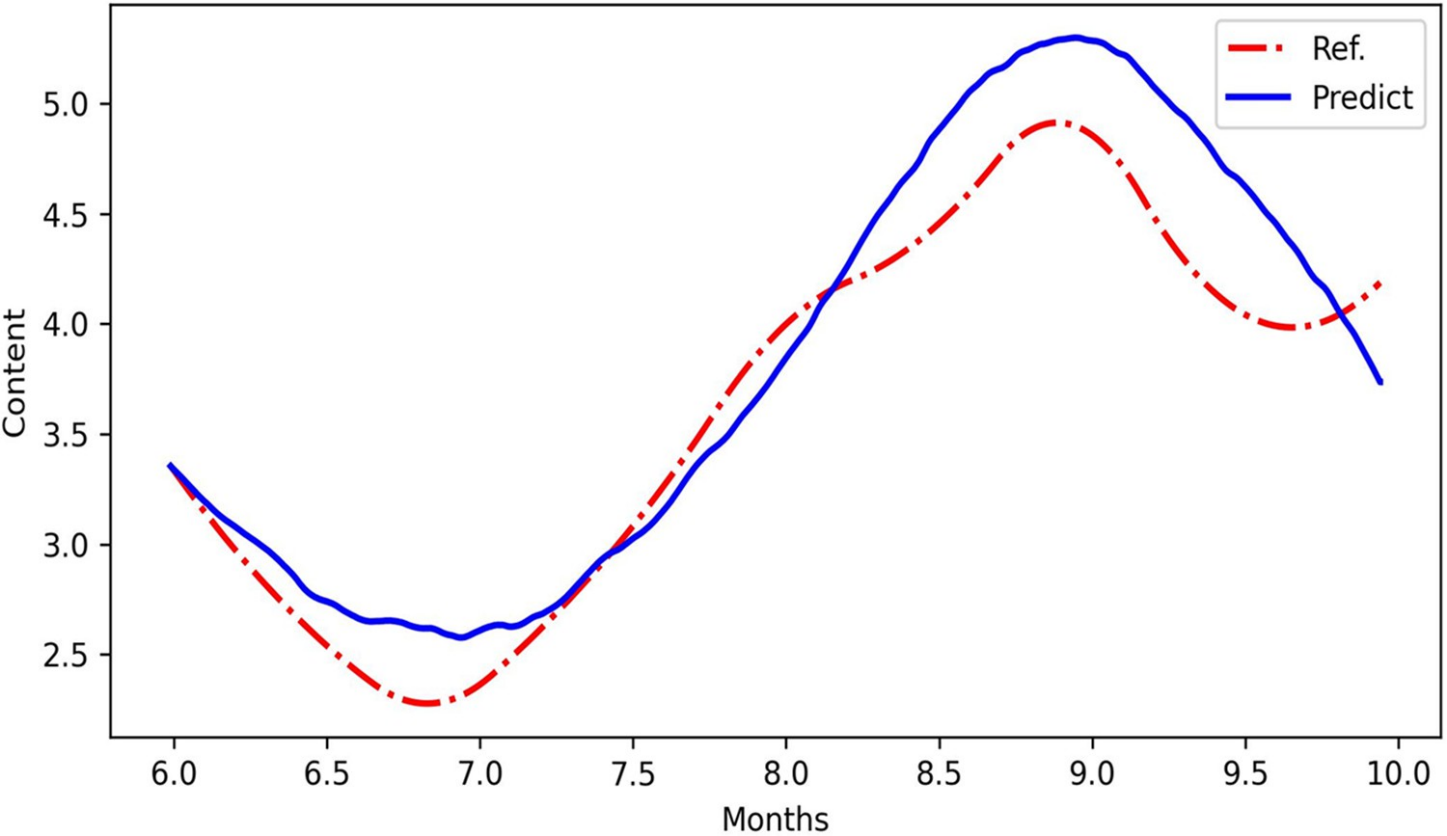
Comparison of predicted (predict) values with reference (ref) values.

A homomorphic white-box neural network was constructed and trained based on the relationship between the environmental factors and secondary metabolite profiles [37], as well as the association among environmental factors, transcriptional regulatory networks, and saponin accumulation. The white-box neural network reflects the quantitative relationship between the environmental factors and secondary metabolites. It retains the regulatory characteristics of the environmental factors and transcriptional regulatory networks that control saponin biosynthesis. The network was trained and loaded with information on the 13 ecological factors to predict the change in saponin accumulation in ginseng of different growth stages (Fig 8 and S1 Fig).

### Model evaluation

**Fig 9:**
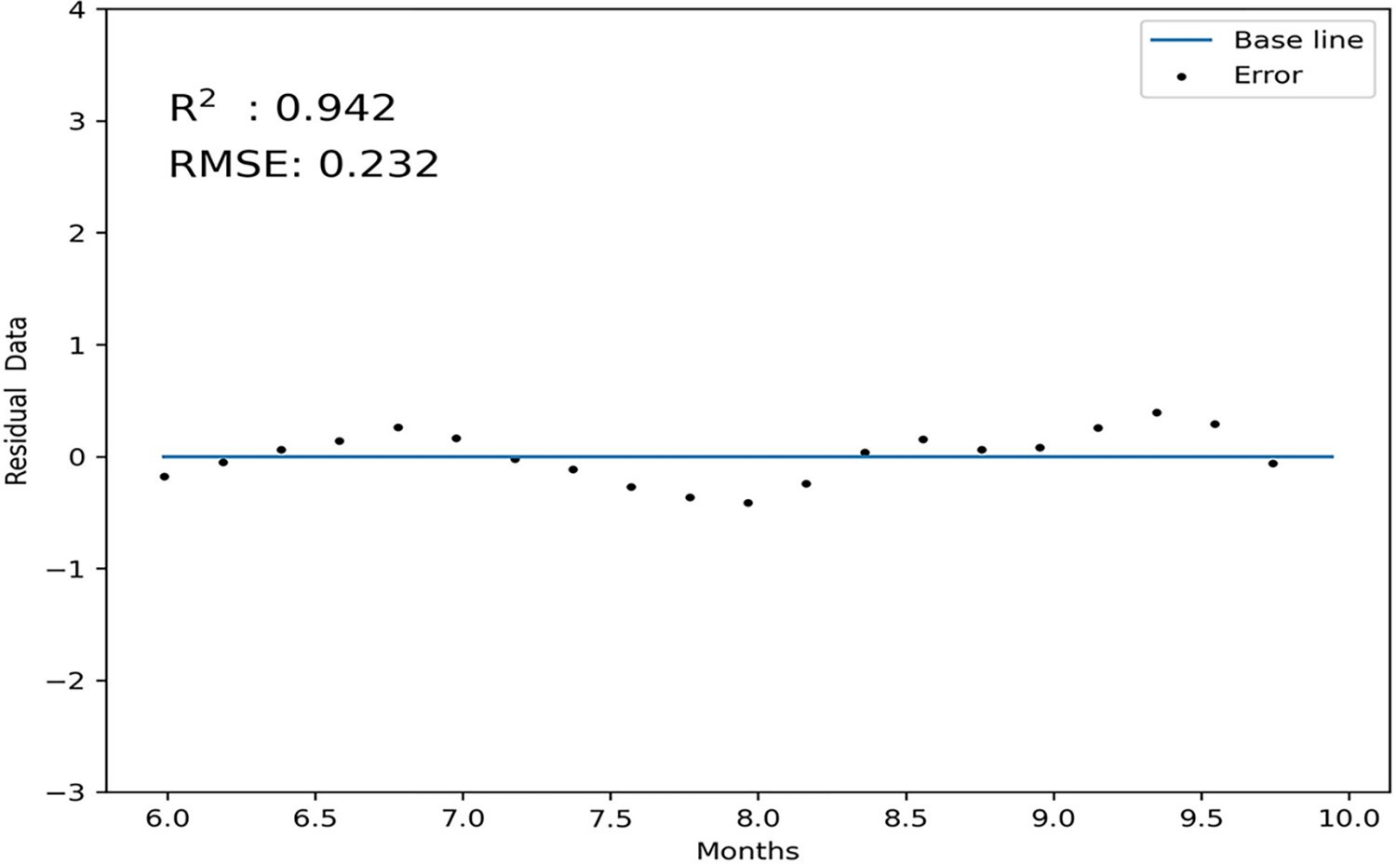
Sample fit of the residual error offset graph.

The predictive performance of the model was evaluated using the residual plot method (Fig 9). The model had an RMSE (root mean square error) of 0.232 and a coefficient of variation of 0.067 (6.7%), which was below the weak variability qualitative standard of 10%. The goodness of fit, R^2^, was 0.942, exceeding the correlation standard of 0.9. These results indicate that the model is reliable with a high accuracy of prediction.

## Discussion

The quality of medicinal herbs is influenced by external environmental factors, and changes in the external environment led to diverse responses in the biosynthesis of medicinal components through transcriptional regulation. Herbal quality is determined by the interplay between the transcriptome and external environmental factors. In line with findings reported by Xu et al. [53], our results indicate that genes involved in saponin synthesis, such as SS, FPS, and DS, are co-expressed. Consistent with the findings of Yang et al., there is a strong correlation between the expression of genes involved in the synthesis of saponin Rb1, including CYP716A47, β-AS, SS, FPS, and DS, and the variation in Rb1 saponin content [54]. The directed graph generated based on the cumulative variance contribution rate and absolute value of the correlation coefficient, which represents the influence of environmental factors on the transcriptome and saponin synthesis, reveals a strong correlation between the contents of essential saponins (Rg1, Re, Rf, Rb1, Rb3, and Rd) and temperature. These results are in accordance with the study by Jia et al. [30], suggesting that within a certain temperature range, the content of ginsenosides decreases with increasing temperature, and moderate low temperature promotes ginsenoside accumulation. Other studies have examined the influence of temperature on ginsenoside accumulation and the expression of key enzyme genes [55], analyzed the biosynthesis of ginsenosides under different water conditions [56], and investigated the impact of soil characteristics on ginseng growth and quality [57]. These studies have highlighted the interplay between ecological factors and ginsenoside synthesis [53]. Furthermore, transcriptome regulation was investigated to identify transcripts that influence ginsenoside synthesis [58]. These studies have provided profound understanding of the regulatory mechanisms of ginsenoside synthesis and its influencing factors. In this study, we analyzed the influence of 13 essential environmental factors on the accumulation of 10 important ginsenosides over time via the regulation of transcriptional networks. Our research adds to the current understanding of the correlation among transcriptional regulation, ecological factors, and ginsenoside accumulation and provides a useful model to interpret such data.

Based on the existing analysis, taking the synthesis of saponin Rb1 as an example, combined with the correlation digraph of the prediction model, the closely related environmental factors were derived from the Rb1 node: Surface temperature, soil temperature, PH, altitude, ground short-wave radiation and soil water potential are the key environmental factors for the synthesis of this saponin. Simulation and regulation of relevant environmental factors, combined with Rb1 synthesis yield index, provide reference for ginsenoside accumulation. At the same time, with the help of this model, TCEMs modules that have an important impact on the synthesis of specific saponins can be obtained, and transcripts that are highly correlated with the eigenvalues of the modules can be examined from such modules, which can provide guidance for the breeding improvement of their related genes by gene editing technology. For example, combined with ginseng reference genome data [55], it can be seen from the correlation among environmental factors, TCEMs and typical saponins that PH value is positively correlated with ME79 and TempBot (Bottom temperature) is positively correlated with ME7 (Fig 2H). The distribution of gene transcripts related to Rb1 saponins at each node of the constructed prediction digraph was investigated. CYP716A53v2, SS, DS and PgUGT71A27 genes were selected to obtain their gene promoter sequence (2000bp). By using plant promoter analysis website PlantPAN3.0 (plantpan.itps.ncku.edu.tw) and JASPAR (jaspar.genereg.net) for a typical promoter element and its active, The highly active promoter element sequences such as AGGAAAT were identified as potential gene editing sites, and the promoter region CAAT-box was considered to mainly control the frequency of transcription initiation and enhance transcription [56]. Demethylation of its associated histones and bases was adopted to improve the activity of the effect of the corresponding promoter element. According to relevant data [57–61], it can be seen that the expression intensity of corresponding genes can be increased by 0.07 to 46.3 times by optimizing the gene editing measures of promoter effect [62]. Considering that ginseng is a diploid plant, the alleles of ginseng genome are treated as above. The above analysis process was applied to the improvement of the prediction model, the association weights of TCEMs nodes and typical saponins Rb1 were modified in combination with the original association relationship of the TCEMs module of the model, and the output weights of ME79 of the coexpression module of the SS gene corresponding transcript were fine-adjusted separately. The prediction results of the model showed that under the conditions of appropriate environmental factors, Rb1 quantity of saponins from 0.02407 $mg\cdot g^{-1}\cdot d^{-1}$ to 0.02498 $mg\cdot g^{-1}\cdot d^{-1}$, daily increases 3.78%, At the same time, the output weight of ME7, the coexpression module of DS gene corresponding to the transcript, was adjusted to increase the amount of saponin Rb1 predicted by the model from 0.02407 $mg\cdot g^{-1}\cdot d^{-1}$ to 0.02707 $mg\cdot g^{-1}\cdot d^{-1}$, an increase of 12.5% per day, and the predicted output was improved. At the same time, gene editing effect of different gene promoter on saponin increase is different, and the validity of this prediction model in breeding and gene improvement of ginseng is also indicated, which provides a reference for practical breeding improvement.

This study acknowledges the broad coverage of environmental factors; however, there is limited representation of soil’s primary and trace nutrient characteristics. The collected ginseng samples were sourced exclusively from authentic production regions. Although soil element characteristics are not the primary stress factors, it is necessary to supplement the study by considering a simulation process that closely aligns with actual conditions. Additionally, it should be noted that different parts of ginseng, such as roots, stems, and leaves, may exhibit variations in the accumulation of secondary metabolites. While this paper considers ginseng as a whole plant, which does not affect the simulation results, further refinement is required in order to account for these differences. The identification of co-expressed transcriptional modules (TCEMs) in ginseng transcriptomes, although rigorously grouped using relevant analysis techniques, requires additional exploration when employing gene editing technologies for breeding improvements. Specifically, investigations focus on co-expressed transcript modules associated with specific saponins, targeting their corresponding gene promoters for simulation-based optimization rather than solely optimizing the expression intensity of specific transcripts. While this approximation holds some rationality, further investigation is necessary to elucidate the biological details underlying these phenomena.

In future research, there is a need to further investigate the potential of digital tools to gain a deeper understanding of secondary metabolite metabolism processes in ginseng and other herbs. The aim is to optimize growth conditions and assist in Chinese herbal breeding practices, particularly with ginseng, to enhance the accumulation of active ingredients like ginsenosides. By doing so, the application of knowledge related to plant growth and development mechanisms in Chinese herbal medicine research will be accelerated. To achieve this, additional efforts should be made to confirm the association between the accumulation of active ingredients in ginseng and the effects of environmental factors. This can be accomplished by studying larger populations and incorporating a broader range of environmental factors. Moreover, understanding the role of transcriptional regulatory networks in mediating these effects will be crucial to fully comprehend the complex interactions between environmental factors and active ingredient accumulation. Through these endeavors, the potential for advancing Chinese herbal medicine research and improving herbal breeding practices, such as with ginseng, will be significantly enhanced, leading to more effective utilization of these valuable medicinal resources.

## Conclusion

This research conducted involved analyzing the correlations among the ginseng transcriptome, 13 essential environmental factors, and 10 important ginsenoside contents. The main objective was to understand the active influence of environmental factors on ginseng biological processes and their direct and indirect correlation with co-expression modules and typical saponin content. To achieve this, a directed graph was constructed to illustrate the influence of environmental factors on saponin accumulation, which was mediated by the transcriptome regulation network. In addition, a white-box neural network was created to train the network, and the validity of this model was verified. The combination of gene ontology analysis, ginseng promoter sequence simulation optimization, and data analysis were utilized based on the gene editing method. This approach helped to validate the practical application of the prediction model in ginseng breeding and improvement. By integrating various factors and using advanced computational methods, this research provides valuable insights into the complex relationship between environmental factors, transcriptome regulation, and ginsenoside content in ginseng. The prediction model developed in this study has the potential to be a useful tool for optimizing ginseng breeding and enhancing the production of important bioactive compounds, such as ginsenosides, to further improve the quality and effectiveness of ginseng in Chinese herbal medicine practices.

## Supporting information

S1 Fig Algorithmic processing flow detailly. (TIF)

S2 Fig Module-environment factors relationships. (TIF)

S3 Fig The correlation coefficient, significance level, and corresponding sample scatter plot between modules and environment factors. (TIF)

S4 Fig Module-saponins trait associations for saponin Rc mainly. (TIF)

S5 Fig Module-saponins trait associations for saponin Rb2 mainly. (TIF)

S6 Fig The correlation coefficient, significance level, and corresponding sample scatter plot between modules and saponins. (TIF)

S1 Table Accurate transcript quantification from RNA-Seq data. (TIF)
