# Supplementary figures and images for: Regulation of transcriptome networks that mediate ginsenoside biosynthesis by essential ecological factors
Source: PLoS One. 2023 Aug 17;18(8):e0290163. doi: 10.1371/journal.pone.0290163 (PMC10434944; doi:10.1371/journal.pone.0290163)

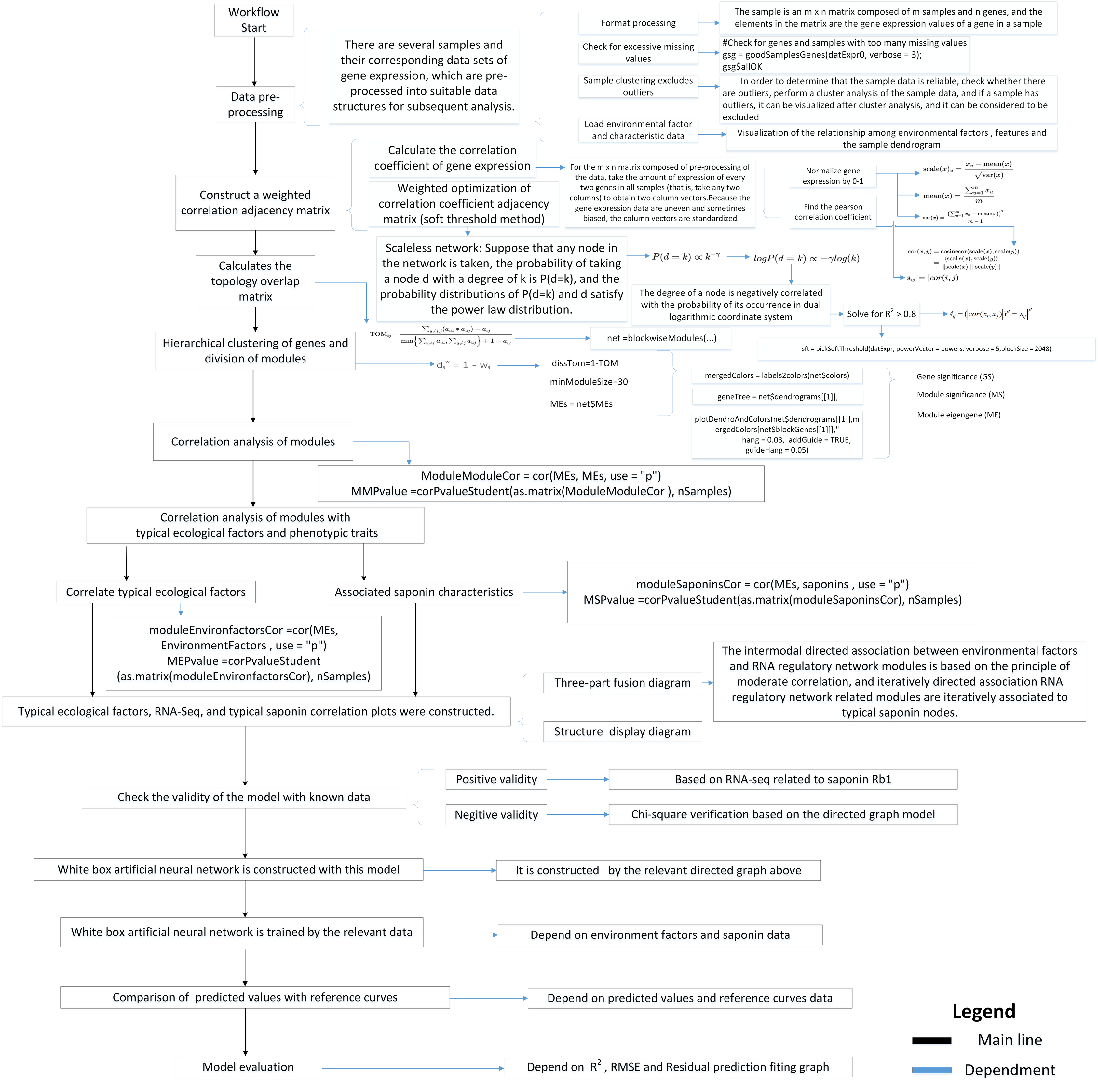

Supplement: S1 Fig — (TIF) [file pone.0290163.s001.tif]

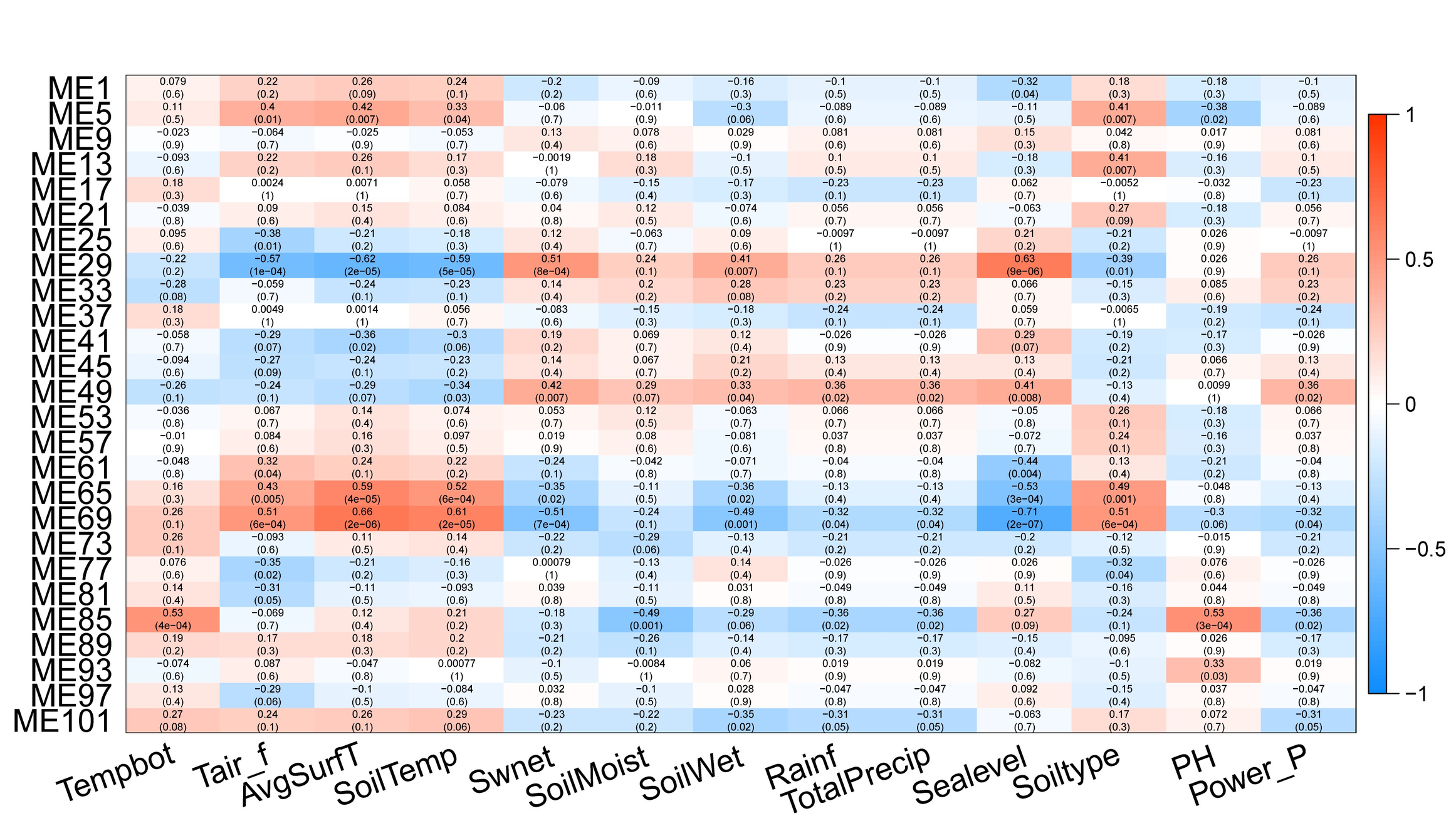

Supplement: S2 Fig — (TIF) [file pone.0290163.s002.tif]

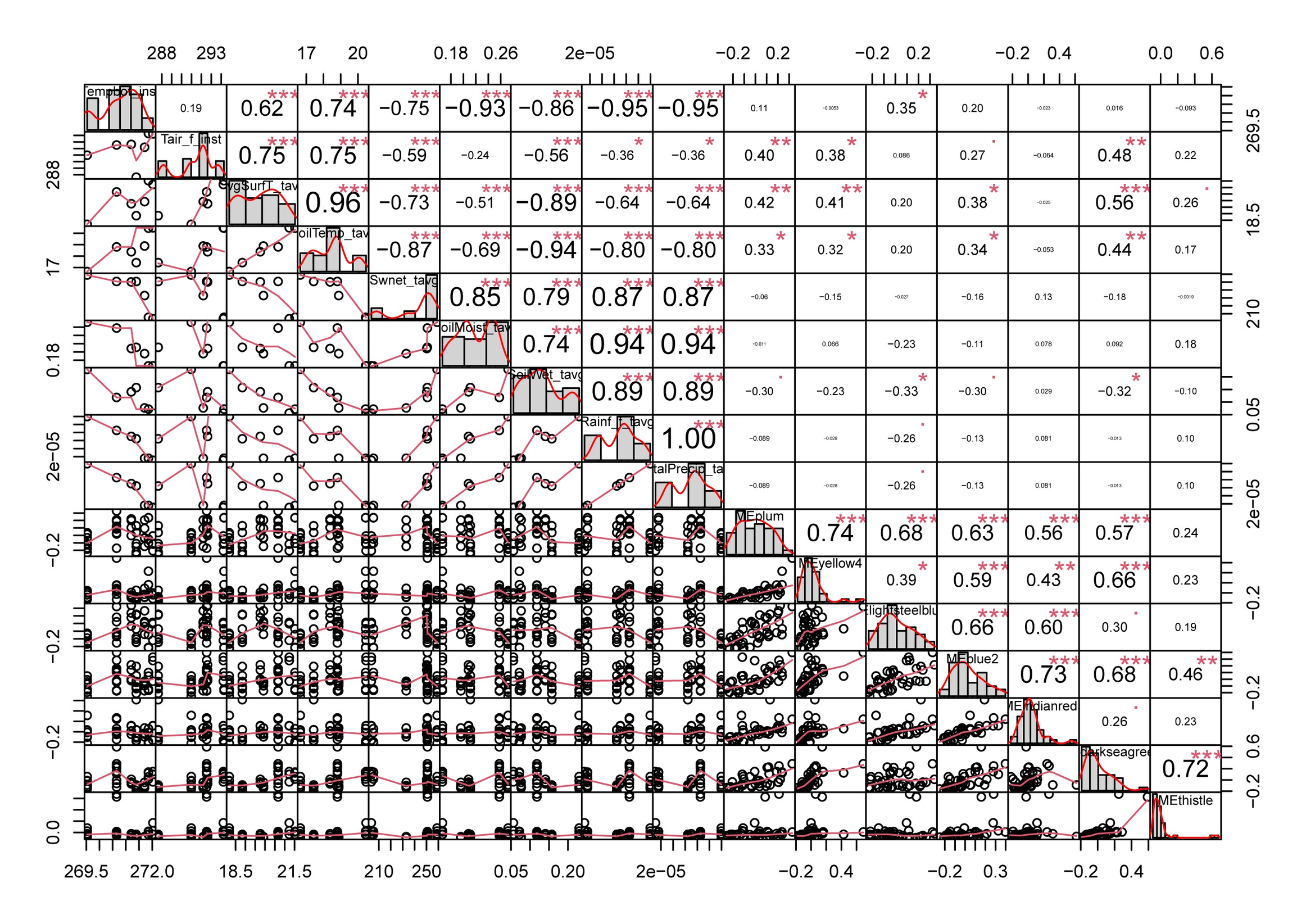

Supplement: S3 Fig — (TIF) [file pone.0290163.s003.tif]

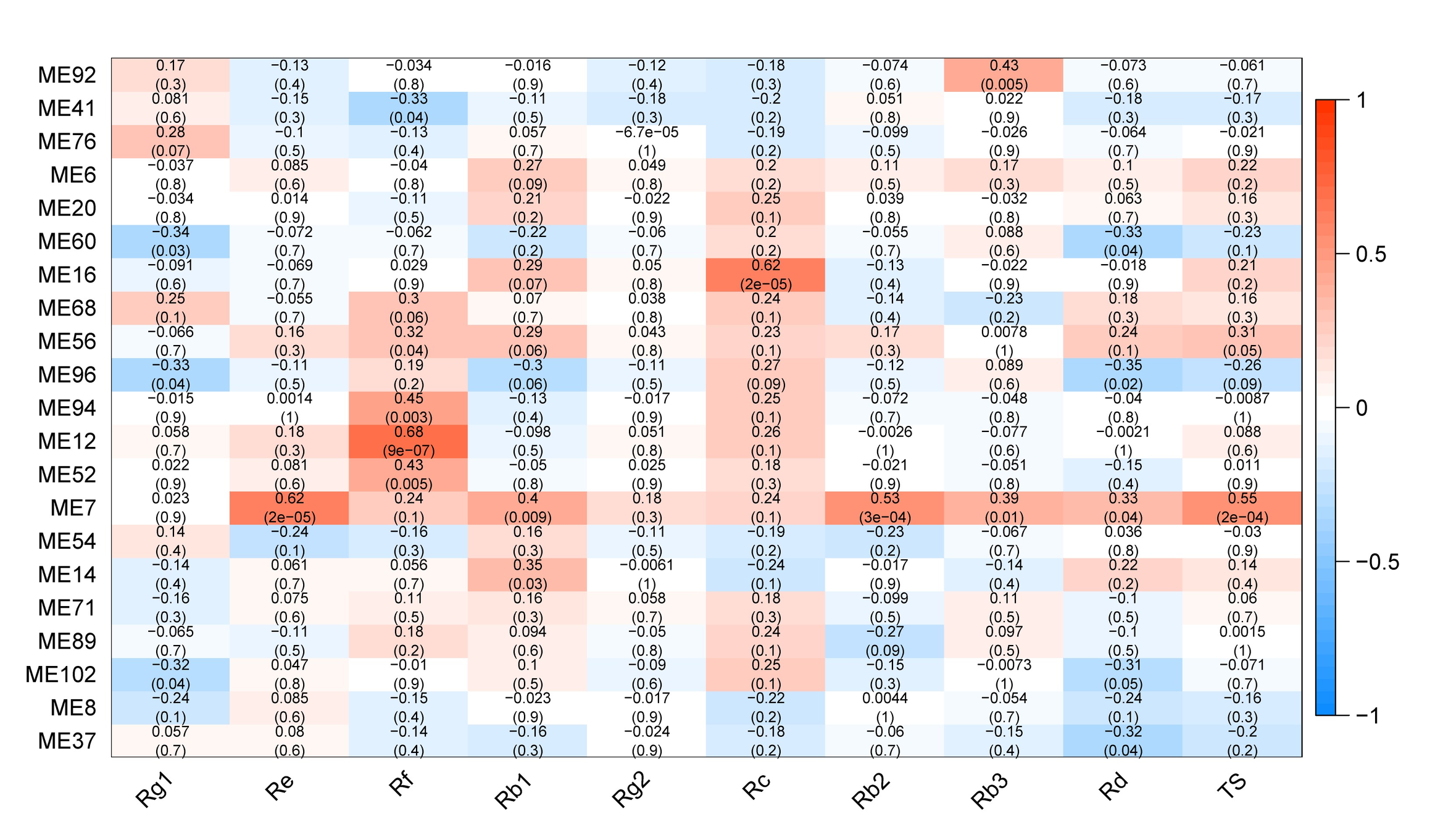

Supplement: S4 Fig — (TIF) [file pone.0290163.s004.tif]

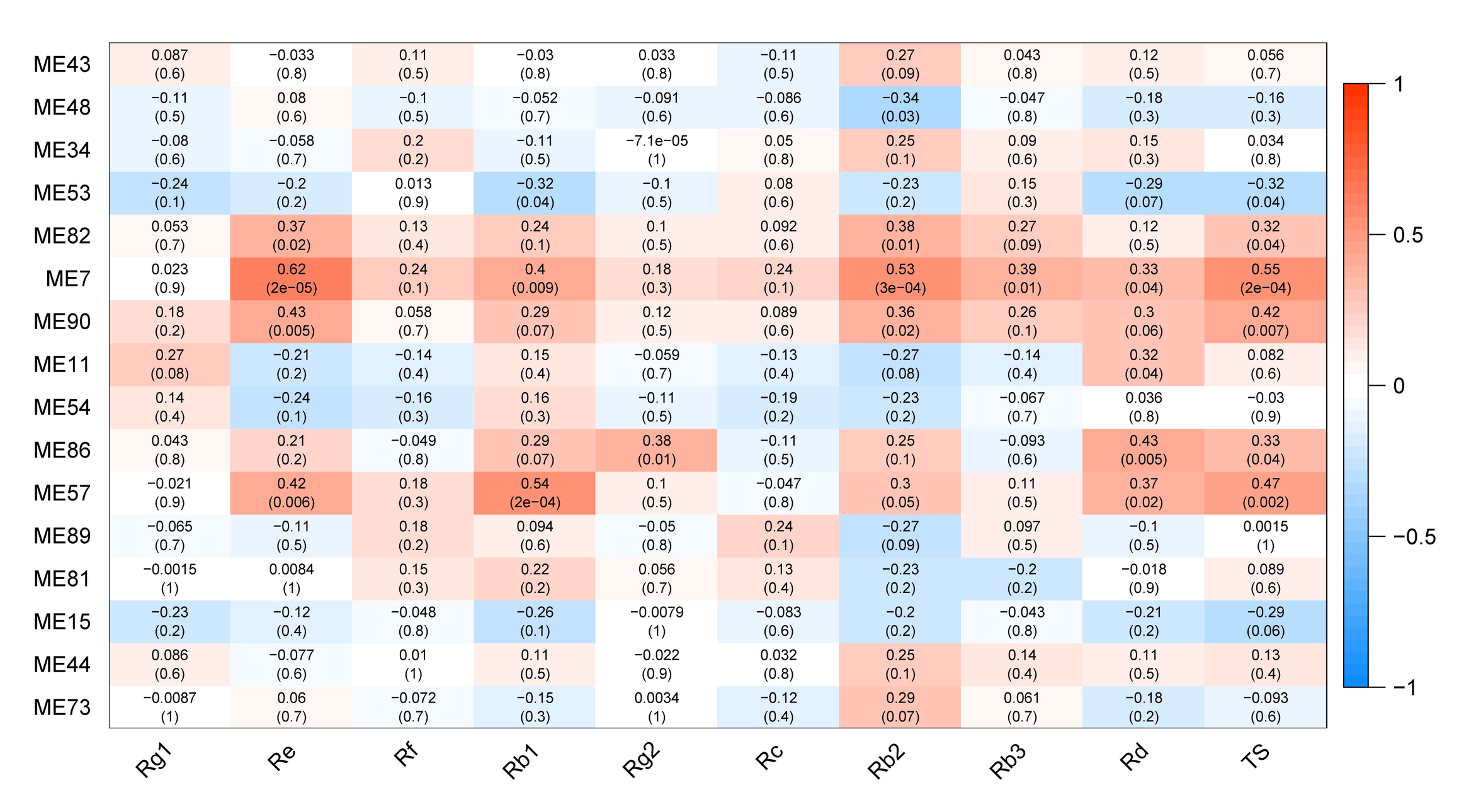

Supplement: S5 Fig — (TIF) [file pone.0290163.s005.tif]

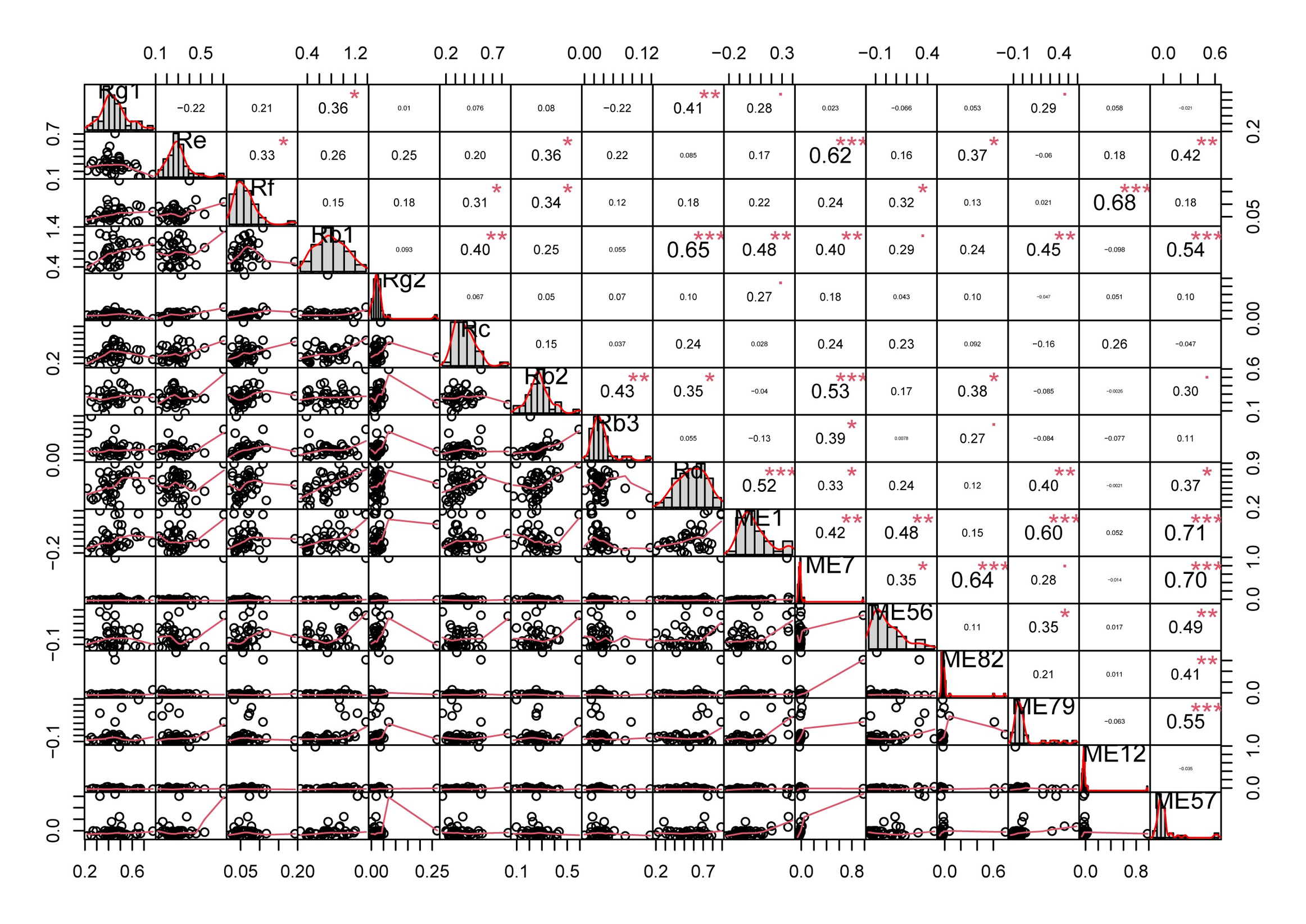

Supplement: S6 Fig — (TIF) [file pone.0290163.s006.tif]

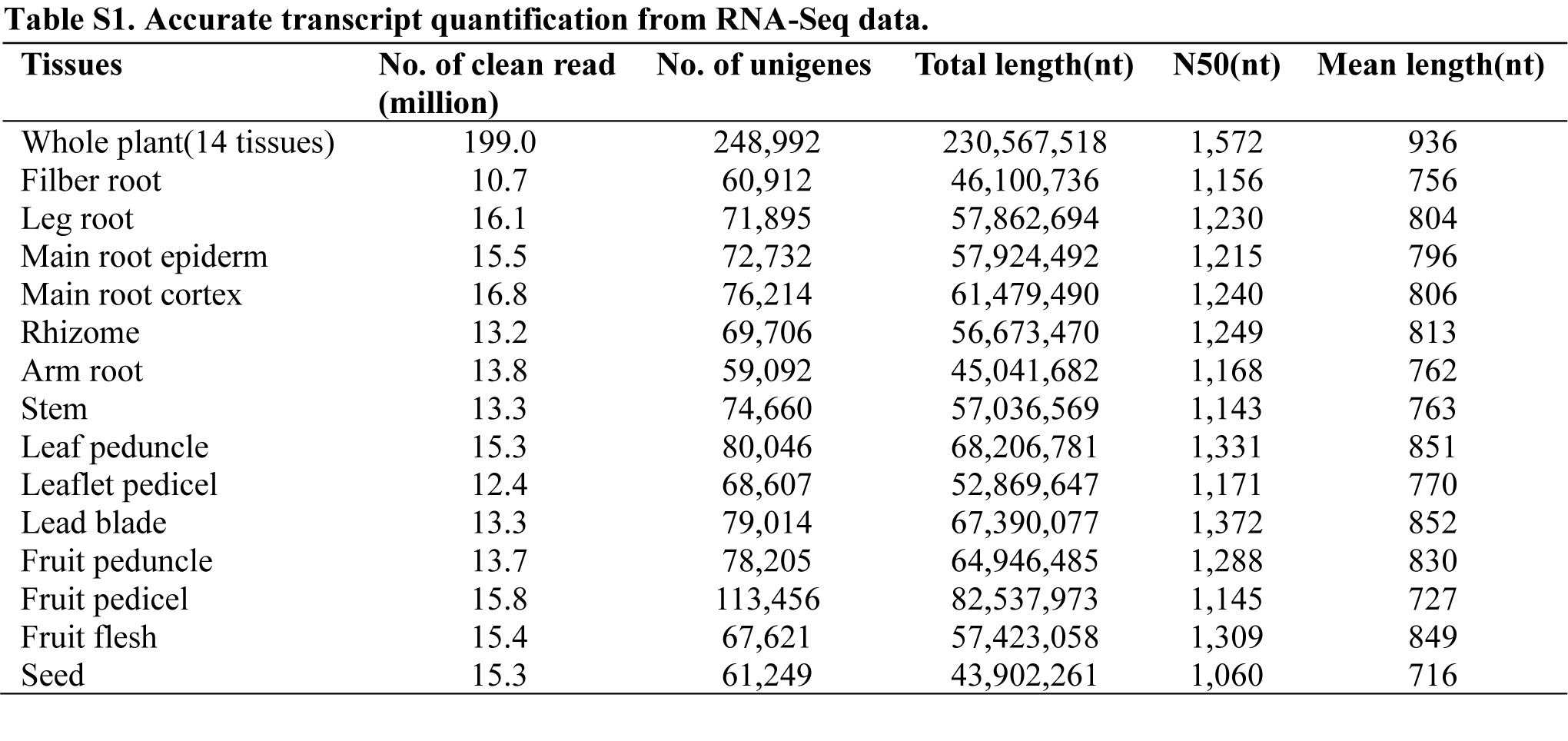

Supplement: S1 Table — (TIF) [file pone.0290163.s007.tif]
